# Supplementary material for: Aerosolized Harmful Algal Bloom Toxin Microcystin-LR Induces Type 1/Type 17 Inflammation of Murine Airways
Source: Toxins (Basel). 2024 Nov 1;16(11):470. doi: 10.3390/toxins16110470 (PMC11598155; doi:10.3390/toxins16110470)
Supplement: Supplementary file 1 [file toxins-16-00470-s001.zip › toxins-3249907-supplementary.pdf]

Supplementary Material

1 Supplementary Data

Table S1. Concentrations of all measured cytokines in the lung lysate.

Protein	Gene Symbol	Description	LLD	LLOQ	Avg. C57BL/6 Male Veh (pg/mL)	Avg. C57BL/6 Male MC-LR (pg/mL)	Avg. C57BL/6 Female Vehicle (pg/mL)	Avg. C57BL/6 Female MC-LR (pg/mL)	Avg. BALB/c Male Veh (pg/mL)	Avg. BALB/c Male MC-LR (pg/mL)	Avg. BALB/c Female Vehicle (pg/mL)	Avg. BALB/c Female MC-LR (pg/mL)
**IL-1a**	*Il1a*	interleukin 1 alpha	0.031	0.19	1.33	4.92	1.77	3.03	9.53	12.98	9.44	9.90
**IL-1b**	*Il1b*	interleukin 1 beta	0.027	0.62	0.45	0.43	0.45	0.47	2.09	1.38	0.99	1.33
**IL-2**	*Il2*	interleukin 2	0.036	0.87	0.98	0.55	0.59	0.79	1.09	0.93	1.15	1.18
**IL-3**	*Il3*	interleukin 3	0.021	0.25	0.21	0.28	0.18	0.37	0.26	0.17	0.28	0.29
**IL-4**	*Il4*	interleukin 4	0.089	0.62	0.27	0.38	0.32	0.38	0.67	0.30	1.06	0.95
**IL-5**	*Il5*	interleukin 5	0	0.65	0.40	0.55	0.61	0.95	0.81	0.65	1.02	0.99
**IL-6**	*Il6*	interleukin 6	0.0066	0.79	0.33	0.51	0.27	0.39	0.29	0.18	0.13	0.21
**IL-10**	*Il10*	interleukin 10	1.09	7.31	0.45	0.65	0.36	0.60	0.19	0.15	0.23	0.22
**IL-12**	*Il12b*	interleukin 12b	0	4.07	3.10	7.22	3.52	7.47	2.38	2.10	2.20	2.30
**IL-17**	*Il17a*	interleukin 17A	0.0089	1.13	0.06	0.36	0.09	0.49	0.33	0.21	0.39	0.30
**MCP-1**	*Ccl2*	chemokine (C-C motif) ligand 2	0.1	11.98	3.61	11.79	3.06	6.29	5.24	5.32	3.52	3.59
**IFNy**	*Ifng*	interferon gamma	0	0.89	0.25	0.49	0.24	0.63	0.60	0.40	0.45	0.46
**TNFa**	*Tnf*	tumor necrosis factor	0.053	0.49	0.13	0.28	0.06	0.16	0.08	0.03	0.08	0.06
**MIP-1a**	*Ccl3*	chemokine (C-C motif) ligand 3	0.0092	0.18	0.38	3.26	0.33	1.77	0.33	0.48	0.17	0.21
**GM-CSF**	*Csf2*	colony stimulating factor 2 (granulocyte-macrophage)	0	0.62	0.41	1.15	0.58	0.99	0.36	0.31	0.27	0.28
**RANTES**	*Ccl5*	chemokine (C-C motif) ligand 5	0.022	0.13	6.94	10.04	7.71	9.89	46.37	18.95	41.81	19.34
**Eotaxin**	*Ccl11*	chemokine (C-C motif) ligand 11	0.087	0.24	46.12	49.84	49.93	51.27	82.45	92.83	65.54	62.92
**MIP-2**	*Cxcl2*	chemokine (C-X-C motif) ligand 2	0.53	22.21	5.61	18.26	6.00	17.03	3.24	2.42	5.34	3.87
**KC**	*Cxcl1*	chemokine (C-X-C motif) ligand 1	0.3	1.56	2.84	15.17	2.14	10.12	8.09	10.90	7.82	8.53
**MDC**	*Ccl22*	chemokine (C-C motif) ligand 22	0.16	6.25	10.10	26.26	12.32	18.88	4.10	5.22	7.51	7.13
**TARC**	*Ccl17*	chemokine (C-C motif) ligand 17	1.37	3.7	292.56	892.63	481.84	577.25	144.76	181.56	235.38	241.51
**TCA-3**	*Ccl1*	chemokine (C-C motif) ligand 1	0.36	0.66	1.53	2.03	1.42	1.98	0.71	0.55	0.18	0.26
**IL-13**	*Il13*	interleukin 13	0.32	10.74	1.41	1.58	1.32	1.60	0.75	0.69	1.22	0.91

Table S2. All lipidomics measurements from lung lysates of male C57BL/6 mice exposed to MC-LR or Vehicle (Veh). All values are in ng analyte per mg total protein.

**Lipid \ Sample ID**	**Veh 1**	**Veh 2**	**Veh 3**	**Veh 4**	**MC-LR 1**	**MC-LR 2**	**MC-LR 3**	**MC-LR 4**	**Avg. Veh Ctrl**	**Avg. MC-LR**
**5(S),15(S)-DiHEPE**	53	11	23	14	255	286	201	226	25	242
**LXB4**	35	10			143	209	223	214	22	197
**19,20-DiHDoPE**	134	52	184	79	336	228	394	253	112	303
**20-COOH LTB4**	7	5		4		42	32	22	5	32
**11,12-DiHETrE**	133	71	257	95	323	575	811	342	139	513
**14,15-DiHETrE**	154	108	468	124	417	850	739	402	213	602

**14-HDoHE**	26	44	49	36	10			18	39	14
**TXB2**		9	23	11		28	26	28	14	27
**13,14dh-15k-PGF2a**	1		2		1	0	1	1	2	1
**15-keto PGE2**	5	15	33	22	4	6	6	4	19	5
**PGD3**	1	2	4	3	0	1	1	1	3	1
**12-HETE**	242	542	466	362	61	281	184		403	175
**9-HODE**	2195	944	1241	495	1708	2736	3528	1964	1219	2484
**12(13)-EpOME**	5	5	5		2	5	3	2	5	3
**5(6)-EpETrE**	1		1	1	1	1	1	1	1	1
**15-epi LXA4**	10		10			70	41		10	55
**12-OxoETE**	198	366	36	188	14		60	24	197	33
**13-HODE**	2673	1544	1515	758	1257	5433	4907	2148	1622	3436
**15(R)-PGE1**			4	3	1	2	2		3	2
**13(14)-EpDPE**	0	0	0	0		0	0	0	0	0
**11(12)-EpETrE**	4	2	3			2	2		3	2
**11(R)-HEDE**	12	4	6	4			16	9	7	12
**13,14dh-15k-PGE2**	12	19	52	27	11	20	16	10	27	14
**15-HETE**	325	160	235	84	166	542	459	239	201	352
**iPF-VI**			0	0			0	0	0	0
**15-OxoEDE**	5	4	1	1		22	7	2	3	10
**12,13-DiHOME**	128	211	523	139	371	1900	466	352	250	772
**5-HETE**	22	18	5	6	3	51	37		13	30
**5,6-DiHETrE**	14	9	71	18	44		74		28	59
**15-OxoETE**			5	5	5	30	27	7	5	17
**9-OxoODE**	893	601	215	293	182	3705	1677	375	501	1485
**16(17)-EpDPE**	0		0	0	0	0	0		0	0

**12(S)-HHTrE**	448	113	174	105	262	762	351	183	210	390
**RvD2**		1	37	1	1	1	1	1	13	1
**PGE1**	4	8	19	9	5	7	8	4	10	6
**Bicyclo PGE2**		1	2	1	0	1	1	1	1	1
**8,9-DiHETrE**	21	17	65	11	28		110	37	28	58
**5-oxoETE**	27	8	4	8	3			2	12	3
**14(15)-EpETrE**	1	1		1	0	0	1		1	1
**13-OxoODE**	1140	1185	285	341	283	8953	2153	373	738	2941
**4-HDoHE**	7	5	1	3	1	22			4	11
**Bicyclo PGE1**	0	0	1	0			0	0	0	0
**15-keto PGF2a**	8	13	34	20	9	19	16	8	19	13
**14(15)-EpETE**	1		1		1		0	1	1	1
**19(20)-EpDPE**	0		0	0	0	0	0	0	0	0
**16-HDoHE**	8	28	14		6	48	36	10	17	25
**PGF1a**	1	1	2	1	1	1	1	1	1	1
**PGE2**	114	200	507	291	161	336	246	136	278	220
**8(9)-EpETrE**	0			0		0		1	0	1
**13(S)-HOTrE**		20	4	3	2			7	9	5
**PGD2**	30	28	79	61	41	60	69		50	57
**9(10)-EpOME**	8	5	8	4		8	2		6	5
**13-HDoHE**	37	8	26	9	31	35		8	20	24
**6kPGF1a**		6	40			16		16	23	16
**5(S),12(S)-DiHETE**	1060		1193	803	602	1152	1076		1019	943
**8-HETE**	10	24	10	6	3		26	15	13	15
**8-isoPGF2a & 11bPGF2a**	2	1	3	1	1	2	2	1	2	1
**13,14dh-15k-PGE1**	0	0	1	0	0	0	0	0	0	0
**PGF2a**	23	12	36	22	22	26	25	15	23	22
**PGJ2**	3	6	13	9	5	11	10	7	8	8

**PGE3**		0	1	1	1	1	1	0	1	1
**13,14dh-15k-PGD2**	22	15	46	19	17	32	31	25	25	26
**20-HDoHE**	14	14	5	6	5		15	8	10	9
**D12-PGJ2**	2		5		2		5		3	3
**PGA2**	5	10	23	15	7	19	16	9	13	13
**LXA5**		10		21				75	15	75
**LTB5**	8		9	8	32				8	32
**20-HETE**	76	64				233			70	233
**9,10-DiHOME**	239	14	831	10			858		273	858
**15-oxo LXA4**		20		11				39	16	39
**5,6-DiHETE(EPA)**	28		20	10			34		20	34
**11-HEPE**			5	2	5				3	5
**9(S)-HOTrE**			1					2	1	2
**LTB4**	271		170			286			221	286
**8(S)-HETrE**				2				2	2	2
**11-HETE**	525		236	152	267				304	267
**RvD1 & AT-RvD1**		1						1	1	1
**tetranor PGEM**				0	0		0		0	0
**PGF3a**	0					0		0	0	0
**10(11)-EpDPE**	1				0				1	0
**2,3-dinor PGE1**	0				0				0	0
**15-keto PGE1**	0	0	1	0			0		0	0
**9-OxoOTrE**		2						1	2	1
**11dh-2,3-dinor TXB2**		0		0				0	0	0
